# Using Zebrafish to Study Collective Cell Migration in Development and Disease

**DOI:** 10.3389/fcell.2018.00083

**Published:** 2018-08-17

**Authors:** Hannah M. Olson, Alex V. Nechiporuk

**Affiliations:** ^1^Department Cell, Developmental & Cancer Biology, The Knight Cancer Institute, Oregon Health & Science University, Portland, OR, United States; ^2^Neuroscience Graduate Program, Oregon Health & Science University, Portland, OR, United States

**Keywords:** collective cell migration, posterior lateral line, posterior lateral line primordium, collective cell invasion, cancer

## Abstract

Cellular migration is necessary for proper embryonic development as well as maintenance of adult health. Cells can migrate individually or in groups in a process known as collective cell migration. Collectively migrating cohorts maintain cell-cell contacts, group polarization, and exhibit coordinated behavior. This mode of migration is important during numerous developmental processes including tracheal branching, blood vessel sprouting, neural crest cell migration and others. In the adult, collective cell migration is important for proper wound healing and is often misappropriated during cancer cell invasion. A variety of genetic model systems are used to examine and define the cellular and molecular mechanisms behind collective cell migration including border cell migration and tracheal branching in *Drosophila melanogaster*, neural crest cell migration in chick and *Xenopus* embryos, and posterior lateral line primordium (pLLP) migration in zebrafish. The pLLP is a group of about 100 cells that begins migrating around 22 hours post-fertilization along the lateral aspect of the trunk of the developing embryo. During migration, clusters of cells are deposited from the trailing end of the pLLP; these ultimately differentiate into mechanosensory organs of the lateral line system. As zebrafish embryos are transparent during early development and the pLLP migrates close to the surface of the skin, this system can be easily visualized and manipulated *in vivo*. These advantages together with the amenity to advance genetic methods make the zebrafish pLLP one of the premier model systems for studying collective cell migration. This review will describe the cellular behaviors and signaling mechanisms of the pLLP and compare the pLLP to collective cell migration in other popular model systems. In addition, we will examine how this type of migration is hijacked by collectively invading cancer cells.

## Introduction

Cellular migration is necessary both during development and adulthood and has been widely studied in populations of cells that migrate independently. However, cells can also migrate in groups in a process known as collective cell migration. During collective cell migration, cells maintain cell-cell contacts, exhibit both morphological and behavioral polarization and interact with neighboring cells within the collective to affect each others behavior. This process is important during the morphogenesis of multiple organ systems, as well as during wound healing in adults. In addition, invading cancer cells exhibit many hallmarks of collective cell migration.

**Figure 1:**
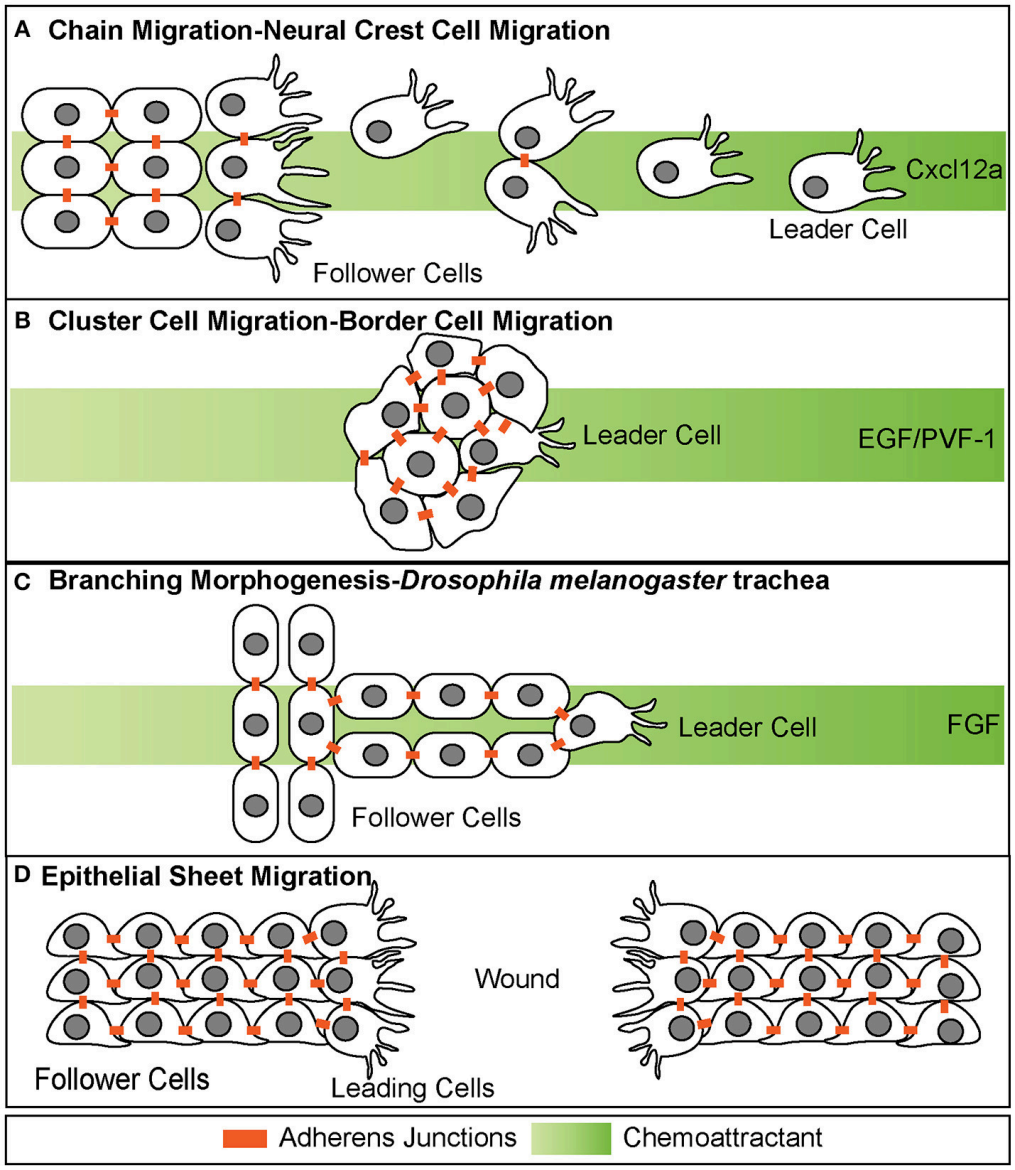
Different modes of collective cell migration. **(A)** Chain migration of neural crest cells. Cells start as a cohesive cluster at the neural plate border and then delaminate away and migrate as chains toward the Cxcl12a source. Cells display transient adherens junctions. **(B)** Cluster cell migration of border cells in *Drosophila melanogaster*. Cells maintain tight adherens junctions while migrating with the leading cell in front exhibiting extensive protrusive behavior. These cells migrate toward the EGF/PVF-1 source. **(C)** Branching morphogenesis of *Drosophila melanogaster* trachea. While the leading cell migrates toward the source of Fgf, trailing cells form tube like structures. **(D)** Epithelial sheet migration-wound healing. Leading cells on either side of the wound migrate toward each other to close the wound. Leading cells extend filopodial protrusions toward each other. Follower cells extend cryptic lamellipodia underneath cells in front of them. Adherens junctions are maintained during migration.

Collectives can be organized in a variety of different forms, including loose chains or strands, tight clusters, tubes, or epithelial sheets (Figure 1). Neural crest cell migration is an example of chain migration (Figure 1A; Rupp and Kulesa, 2007). During migration, neural crest cells migrate out the neural tube in a chain like manner with the ultimate goal of reaching distant sites and differentiating into numerous cell types (Theveneau and Mayor, 2012). Throughout migration, neural crest cells maintain transient adherens junctions when briefly in contact with each other. Specifically, when two migrating neural crest cells touch, they induce contact inhibition of locomotion (Mayor and Carmona-Fontaine, 2010). This causes the two cells to retract cellular extensions at the site of contact and initiate new extensions on the opposing side of contact of the cell. This behavior restricts protrusions within the interior portion of the chain of migrating cells and promotes protrusive behavior along the edges of the chain, specifically the leading edge (Carmona-Fontaine et al., 2008). This behavior is thought to be important for directional migration and self-organization, and loss of this behavior has been shown in invasive cancer cells (Carmona-Fontaine et al., 2008; Astin et al., 2010).

**Figure 2:**
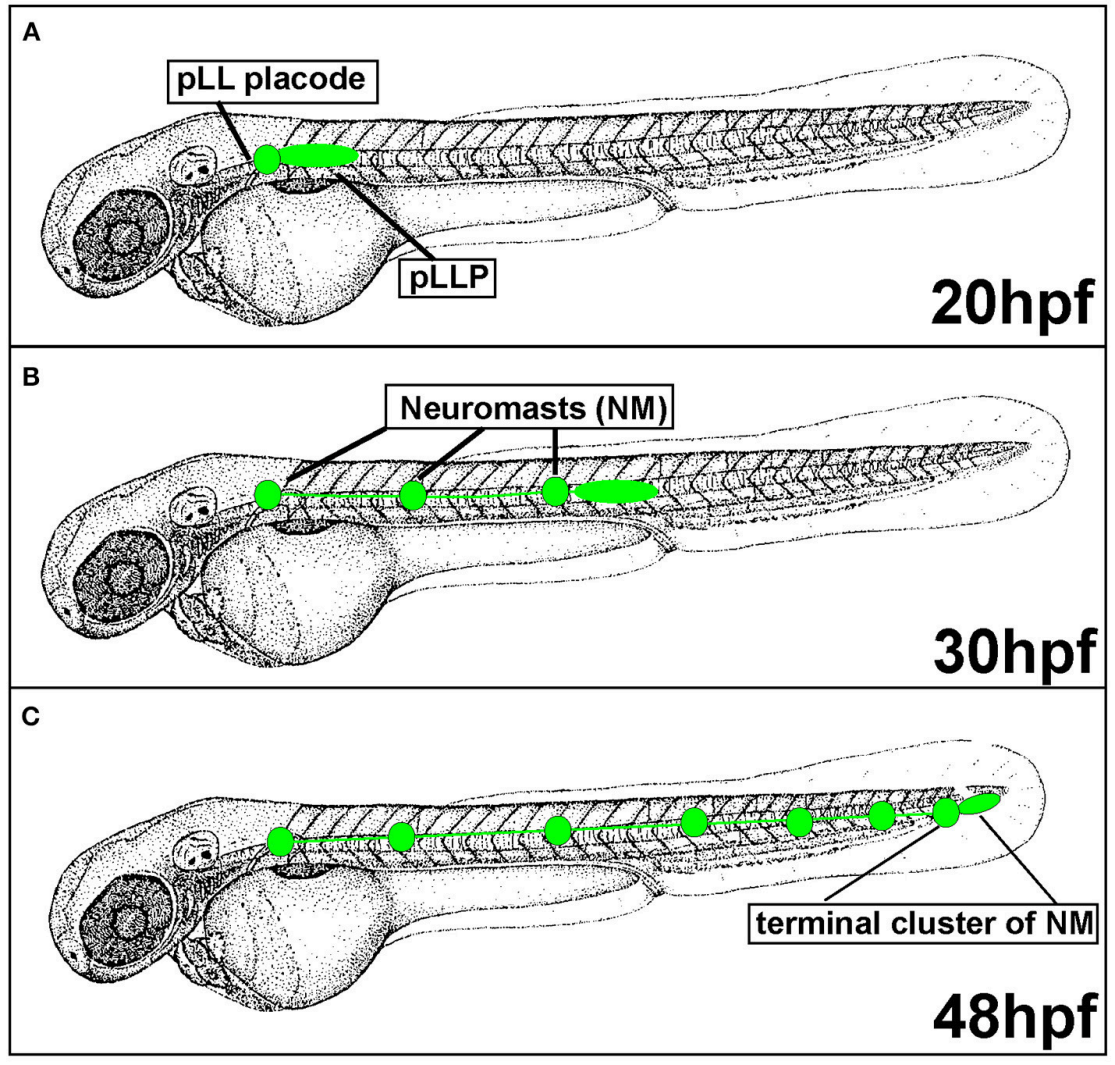
Posterior Lateral Line formation (pLL) and posterior Lateral Line Primordium (pLLP) migration. **(A)** pLLP begins migrating around 20 hours post-fertilization (hpf). **(B)** At 30 hpf the pLLP has migrated about half way down the trunk and deposited 3 neuromasts (NMs). **(C)** pLLP migration is complete at 48 hpf with the deposition of the terminal cluster of NMs.

**Figure 3:**
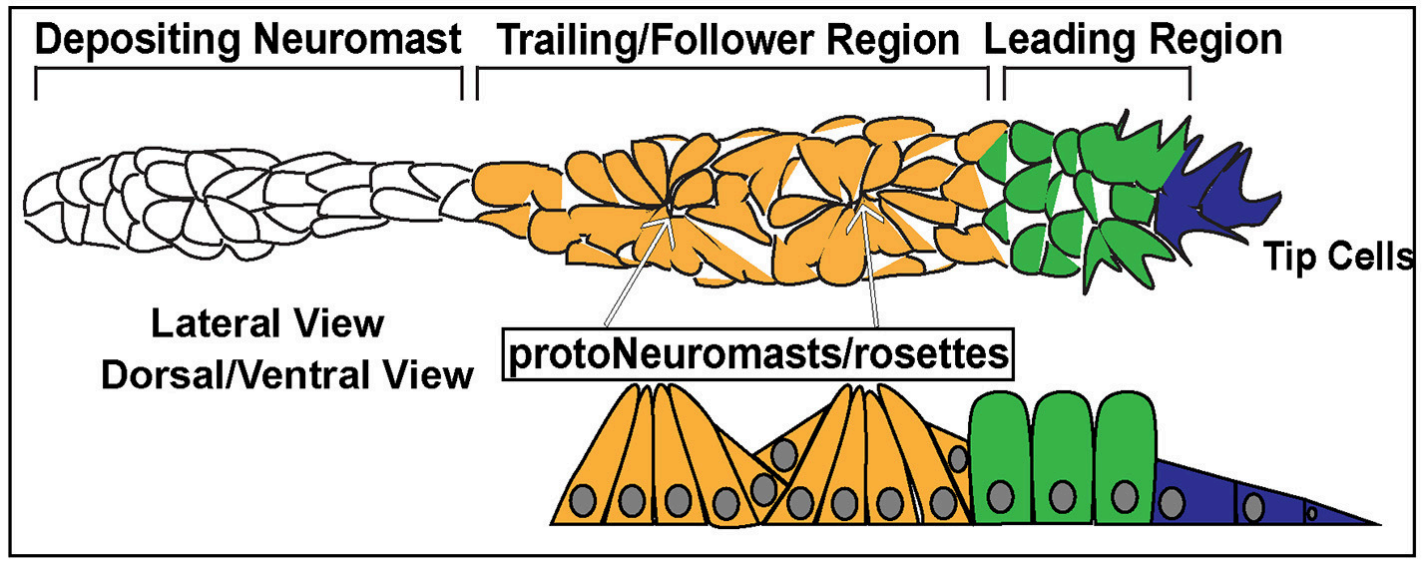
Schematic of the posterior Lateral Line Primordium (pLLP). In blue are the 2–3 leader cells. In green is the leading region. In orange is the trailing region. In white is a depositing neuromast. The top schematic is a lateral view of pLLP cells. The bottom schematic is a dorsal/ventral view. Arrows point to proto-neuromasts/rosettes.

Collectives can also migrate in a much more cohesive group, often referred to as cluster cell migration (Figures 1B, 2). During migration of this type, cells maintain adherens junctions while migrating, thus remaining tightly connected. Examples of cluster cell migration include pLLP migration in zebrafish (Figures 2, 3), border cell migration in *D.melanogaster* (Figure 1B), Kupffer vesicle organogenesis in zebrafish, and movement of invasive clusters of tumor cells (**Figure 6**). During border cell migration a group of cells delaminates from the follicular epithelium of the *D.melanogaster* egg chamber and migrates across the chamber toward the developing oocyte (Montell et al., 1992). During this migratory process, these cells maintain adherens junctions while migrating. Similarly, during Kupffer vesicle organogenesis, a group of around 20-30 cells cluster together and migrate cohesively. Finally, invasive groups of tumor cells often migrate as clusters during the invasion of many epithelial-based tumors (**Figure 6**; Freidl et al., 2004; Christiansen and Rajasekaran, 2006; Alexander et al., 2008). This type of collective invasion is discussed in more detail below.

Collective cell migration also contributes to the process of branching morphogenesis. This cellular behavior drives the formation of highly branched tubular structures including mammalian kidneys, lung, prostate and mammary gland as well as *D.melanogaster* trachea (Figure 1C; Sutherland et al., 1996; Ewald et al., 2008; Metzger et al., 2008). During branching morphogenesis, epithelial sheets reorganize into tube-like structures through multiple cellular behaviors, one of which is a specialized type of collective cell migration called invasive branching. During this process, extension of new branches is guided through invasive migratory behavior of a tip cell, which exhibits dynamic protrusive behavior. The cells that lag behind the tip cell are referred to as “stalk cells” and maintain cadherin-mediated adhesion to each other as well as to the tip cell.

In contrast to branching morphogenesis, epithelial sheet migration involves collective movement of a leading cell front, rather than individual cells (Figure 1D). Epithelial sheet migration mediates wound healing in the adult, dorsal closure in *D. melanogaster*, and migration of germ layers in some animals (Martin and Parkhurst, 2004; Solnica-Krezel, 2005). This type of collective cell migration is also observed *in vitro* after a scratch wound assay of endothelial and epithelial cells Figure 1D. In this assay, a scratch is made across a confluent sheet of cells and sheet migration is observed to “heal” or repair the “wound,” as cells migrate as a cohesive front to fill in the space that was created by the scratch. On a cellular level, sheet migration is characterized as a monolayer of migrating cells that maintain strong adherens and tight junctions as well as apico-basal polarity while migrating (Bahri et al., 2010). These junctions restrict movement within the sheet and limit cellular rearrangement (Zallen and Blankenship, 2008). Cells at the leading or “free edge” take on leader cell positions and extend actin-based cellular protrusions (Figure 1D; Poujade et al., 2007; Vitorino and Meyer, 2008; Omelchenko et al., 2014). Follower cells also exhibit protrusive behavior through the extension of cryptic lamellipodia, as seen during wound closure in MDCK cells *in vitro* (Figure 1D; Fenteany et al., 2000; Farooqui and Fenteany, 2005). These cryptic lamellipodia form at the basal side of the follower cells and protrude under cells in front. These lamellipodia are necessary for generating traction against the basal lamina (Fenteany et al., 2000; Farooqui and Fenteany, 2005).

From these examples, it is clear that collective cell migration is employed during the morphogenesis of multiple organ systems. Despite this diversity, collectives often employ conserved cellular strategies during the migratory process. Our understanding of these mechanisms comes from studying various models including pLLP migration. Here, we will first review the cellular and molecular mechanisms that drive pLLP migration. Then we will briefly review a few other examples of collective cell migration and compare the pLLP to these other forms of collective cell migration. Finally, we will discuss how mechanisms of collective cell migration can be misappropriated during cancer cell invasion.

### Zebrafish posterior lateral line primordium as a model system to understand collective cell migration

Over the years a variety of model systems have been used to understand cellular and molecular mechanisms of collective cell migration. These range from the slime mold *Dictyostelium discoideum* to study movement of cell aggregates, to the mouse retina to investigate blood vessel branching. With zebrafish emerging as a genetic model system in the 1980s and 1990s, collective cell migration of the lateral line primordium during embryogenesis became one of the premier model systems to dissect collective cell migration. Lateral line research has a rich history, as studies examining this system date back to the 1600s, when scholars first discovered that various fish species contain a row of small pores that stretch along the trunk of the fish from the head to the tail. At that time it was believed that this was a glandular system required for the secretion of mucous that covered the fish (Monro, 1785). However, in the 1800's that view was challenged when it discovered that this system is in fact a mechanosensory system, similar to the touch sensory system within the skin (Knox, 1825). Around 1850, Leydig discovered that within the row itself there were actually small sensory organs and in 1870, Schulze postulated that these sensory organs were actually similar to those within the inner ear and that movement of water stimulated their sensory capabilities (Leydig, 1850; Schulze, 1861, 1870). Up until this point however, the majority of research had focused on observing the system but not perturbing it. Fuchs (1894) was one of the first scholars to experimentally test the system and discovered that the lateral line in *Torpedo* only responded to changes in tactile stimulation but not in regards to changes in temperature or chemical stimulation (Fuchs, 1894). Finally, in 1904 Parker took a systematic approach to examine factors that stimulated lateral line sensory organs in eight different species of fish (Parker, 1904). He tested light, heat, salinity, food, oxygen, carbon dioxide, foulness of water, water current, water pressure, high frequency vibrations (hearing), and low frequency vibrations. Interestingly, he found that only vibrations of low frequency were sensed by the lateral line. He concluded that the lateral line system was a mix of the touch (skin) sensory system and the hearing (ear) sensory system. Further studies confirmed these findings and expanded on them culminating in the conclusion that the lateral line in aquatic vertebrates is a mechanosensory system that detects changes in water current and is necessary for behaviors such as feeding and swimming (Montgomery et al., 2000).

Harrison (1904) was the first to determine that this mechanosensory system develops through the migration of a group of cells that deposits smaller clusters of cells while migrating (Harrison, 1904). Specifically, Harrison used chimeric frog embryos to study posterior lateral line (pLL) development. In this study Harrison fused the head of a black tadpole to the tail of brown tadpole and observed pLL development. Surprisingly, he witnessed a dark streak (black cells) appear on the brown tadpoles tail and this streak separated into pigmented dots. From this he concluded that the lateral line develops by the concerted migration of a group of cells down the trunk of fish and amphibians. Further research in the 1920s and 1930s identified the origin of this group of cells. Stone (1933) discovered that this group of cells originates from the post auditory placode (Stone, 1933). Specifically, Stone stained salamander embryos with Nile blue sulfate and then grafted the post auditory placode from these stained donor embryos to unstained host embryos. Following transplantation he observed a group of blue stained cells migrating along the trunk of the embryo that deposited small clusters of blue stained cells (presumptive mechanosensory organs—neuromasts). These clusters then differentiated into the sensory organs that form the lateral line. These experiments identified the post auditory placode as the group of cells giving rise to the pLL. As evident from these classical experiments, the close proximity to the surface of the skin makes this an easily tractable system to study mechanisms of mechanosensation and collective cell migration.

### Posterior lateral line development and posterior lateral line primordium migration

Since the pLLP discovery in the early twentieth century, the migratory behavior of this system has been actively investigated in various aquatic species including zebrafish. In zebrafish, the posterior Lateral Line Primordium (pLLP) is a group of around 100 cells that migrates along the lateral aspect of the trunk of the embryo during embryogenesis (Figure 2). The pLLP and sensory neurons of the pLL ganglion are both derived from the pLL placode, a transient thickening of the embryonic ectoderm positioned caudally to the developing otic vesicle (Figure 2; Mizoguchi et al., 2011). Surprisingly, little is known about molecular pathways that regulate pLL placode induction and differentiation and this topic has been discussed elsewhere (Sarrazin et al., 2010; McCarroll et al., 2012; Piotrowski and Baker, 2014; Nikaido et al., 2017). At about 22 hours post fertilization (hpf), the distal portion of the pLL placode begins migrating along the lateral aspect of the trunk, whereas the proximal portion, comprised of sensory neurons, stays behind (Figure 2A). The pLLP continues migrating along the trunk of the zebrafish until it reaches the tip of the tail at 48 hpf (Figure 2C). As the pLLP migrates, it deposits clusters of about 20–30 cells from its trailing (caudal) end; these clusters will differentiate into mechanosensory neuromasts (NMs) (Figures 2, 3). The pLLP also lays down a single line of inter-neuromast cells (Figure 2; Metcalfe et al., 1985), which are latent precursors that will differentiate into additional NMs during larval development. Migration of the pLLP is complete with the deposition of the terminal cluster, a group of two to three NMs that are located at the distal region of the trunk (Figure 2C). Rapid embryonic development, optical translucence, and genetic tractability make the zebrafish a particularly suitable model system to define cellular and molecular mechanisms of collective cell migration.

### How is the posterior lateral line primordium organized?

Cells within the pLLP display differential morphology and different states of differentiation depending on their location and can be generally designated as leader or follower cells. The leading third of the pLLP is comprised of 2–3 tip cells of mesenchymal character and less differentiated epithelial cells (Figure 3, Blue, Green). Follower cells within the trailing two-thirds of the pLLP (Figure 3, Yellow) form polarized rosettes. Tip cells exhibit flat mesenchymal morphology (Figure 3), display active protrusive behavior in their leading edge, and respond to guidance cues that steer the collective. Cells proximal to tip cells in the leading region, exhibit columnar epithelial morphology (Figure 3). Cells within the trailing region (last 2/3 of the pLLP) apically constrict to form epithelial rosette structures of the proto-NM (Figure 3; Lecaudey et al., 2008). The remaining cells in the trailing region contribute to the inter-neuromast cells and are deposited between the NMs. These cells are positioned on the periphery of the pLLP, surrounding the cells that have formed rosettes (Dalle Nogare et al., 2017).

In addition to differences in morphology, leader and follower cells within the pLLP show differences in their fate. As the pLLP migrates it deposits proto-NMs every 5–7 somites. At the onset of migration the pLLP contains 2 to 3 proto-NMs; however, by the end of migration the pLL consists of 5–6 NMs and the terminal cluster of NMs (Figure 2). Thus, new proto-NMs must be generated during pLLP migration. These new proto-NMs are generated by cell proliferation throughout the pLLP (Dalle Nogare et al., 2017). Newly generated cells within the leading region of the pLLP differentiate last and ultimately contribute to the terminal cluster of NMs, while those in the trailing region begin differentiating into cells that will contribute to more proximal NMs and inter-neuromast cells (Dalle Nogare et al., 2017). As a new proto-NM begins differentiating, cells undergo apical constriction to form a rosette-like structure that constitutes the proto-NM (Figure 3).

### Chemokine signaling during posterior lateral line primordium migration

**Figure 4:**
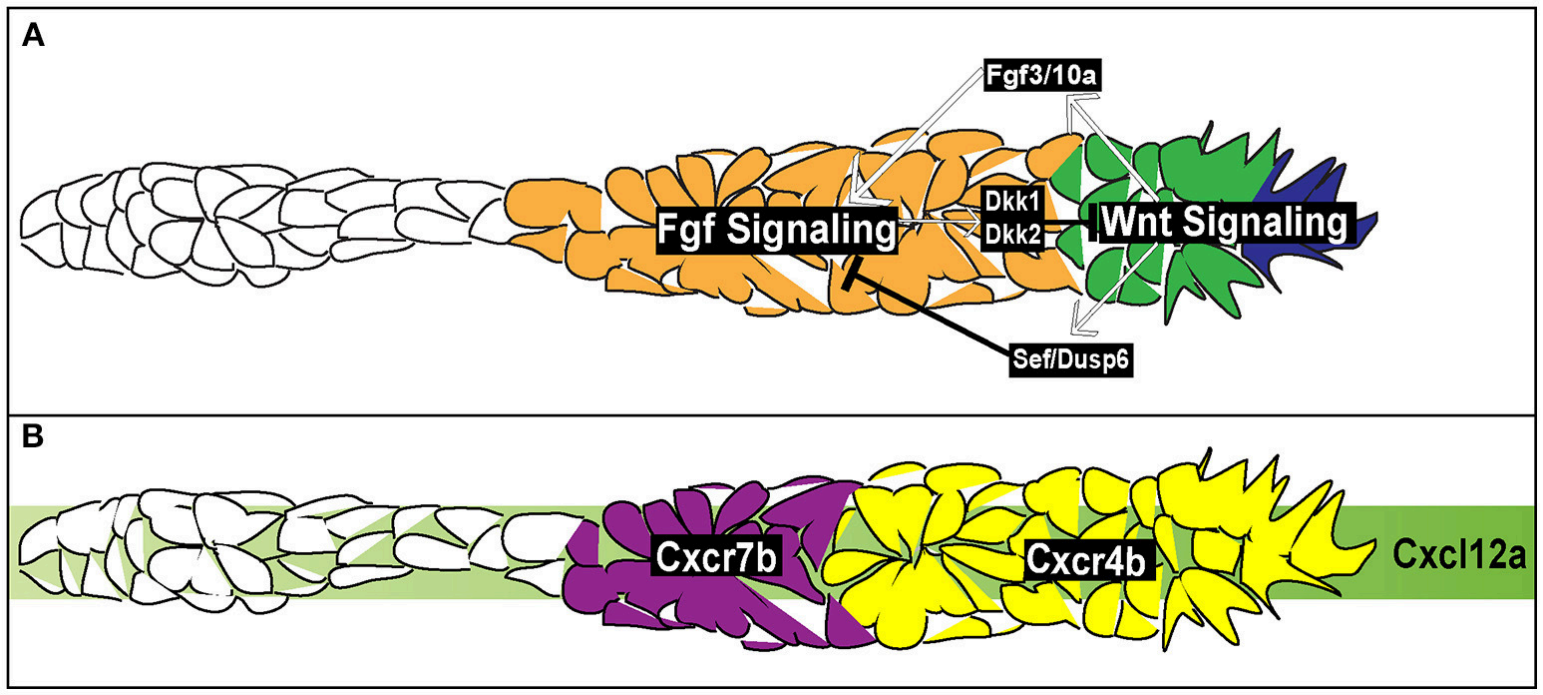
Signaling and chemotactic pathways active during pLLP migration. **(A)** Signaling pathways active during pLLP migration. Wnt signaling is active in the leading region (blue and green cells). Wnt signaling initiates expression of Fgf3/10a in the leading cells. Fgf 3/10a activate Fgf signaling in the trailing region (orange). Wnt signaling initiates expression of *sef* , an inhibitor of Fgf signaling. Fgf signaling initiates expression of *dkk1* and *dkk2*, inhibitors of Wnt signaling. Thus the two signaling regions are maintained through mutual inhibition. **(B)** Chemokine signaling during pLLP migration. Green strip indicates the internal Cxlc12a generated within the pLLP. *cxcr4b* chemokine receptor (yellow) is expressed in the leading 2/3 of the pLLP. *cxcr7b* is expressed in the trailing 1/3 of the pLLP (purple).

Similarly to neural crest cell migration, the pLLP uses Cxcl12a as a chemotactic cue. The pLLP migrates along the myoseptum of the zebrafish embryo where *cxcl12a* is uniformly expressed (Figure 4B; David et al., 2002; Li et al., 2004; Haas and Gilmour, 2006; Dambly-Chaudiere et al., 2007; Valentin et al., 2007). Loss of Cxcl12a leads to a failure of migration (David et al., 2002; Valentin et al., 2007), whereas ectopic expression of *cxcl12a* results in a redirection of pLLP toward the “new” Cxcl12a source (Li et al., 2004).

While pLLP can migrate toward an ectopic source of the ligand, two recent studies demonstrated that the Cxcl12a does not present as a gradient along the trunk (Donà et al., 2013; Venkiteswaran et al., 2013). Instead, the pLLP produces an internal gradient of Cxcl12a through differential expression of two chemokine receptors, *cxcr4b* and *cxcr7b*. *cxcr4b* is expressed within the leading region whereas *cxcr7b* is expressed within the trailing region (Figure 4B; Haas and Gilmour, 2006; Dambly-Chaudiere et al., 2007; Valentin et al., 2007). Loss of either Cxcr4b or Cxcr7b leads to a failure in migration (Haas and Gilmour, 2006; Valentin et al., 2007) indicating the necessity of both receptors to ensure proper pLLP migration. Additionally, wild-type cells transplanted to the leading region of *cxcr4b* mutant pLLP can rescue migratory defects in these mutants (Haas and Gilmour, 2006). This is also true for wild-type cells transplanted to the trailing region of *cxcr7b* mutants (Valentin et al., 2007). However, when wild-type cells are transplanted to the leading region in *cxcr4b* mutants, impaired migration is not rescued. Finally, when *cxcr7b* mutant cells are transplanted into the leading region of *cxcr4b* mutants, migration is rescued (Valentin et al., 2007). Altogether, these transplantation experiments underscore the necessity for region-specific distribution of Cxcr4b and Cxcr7b during pLLP migration. Two recent studies used live imaging to visualize chemokine-receptor internalization to demonstrate that Cxcl12a binds to Cxcr7b and then is internalized with the Cxcr7b receptor (Donà et al., 2013; Venkiteswaran et al., 2013). In doing so, this creates an internal gradient of Cxcl12a, with low levels of Cxcl12a in the trailing region and high levels of Cxcl12a in the leading region. Previous reports support this model as Cxcr7b acts as a ligand sink in other contexts (Dambly-Chaudiere et al., 2007; Boldajipour et al., 2008; Naumann et al., 2010; Mahabaleshwar et al., 2012).

At this point, it is unclear how this region specific expression of *cxcr4b* and *cxcr7b* in the leading and trailing region arises. Aman and Piotrowski (2008) argued that *cxcr7b* expression is downstream of two signaling pathways active within the pLLP, Wnt (leading region) and Fgf (trailing region) signaling (Figure 4A). When Wnt was constitutively active or Fgf was inhibited there was a reduction in *cxcr7b* expression. Additionally, while inhibition of Wnt signaling had no effect of *cxcr4b* expression it did lead to an expansion of *cxcr7b* expression into the leading region. However, a separate study did not report any effect on *cxcr4b* or *cxcr7b* expression in the absence of Wnt signaling (Valdivia et al., 2011). It should be also noted that expression of chemokine receptors does not mirror Wnt and Fgf signaling domains, suggesting that these receptors are not directly regulated by these signaling pathways (Figure 4). Further experiments are needed to determine how chemokine receptor expression is regulated during pLLP migration.

### Signaling pathways within the posterior lateral line primordium

**Figure 5:**
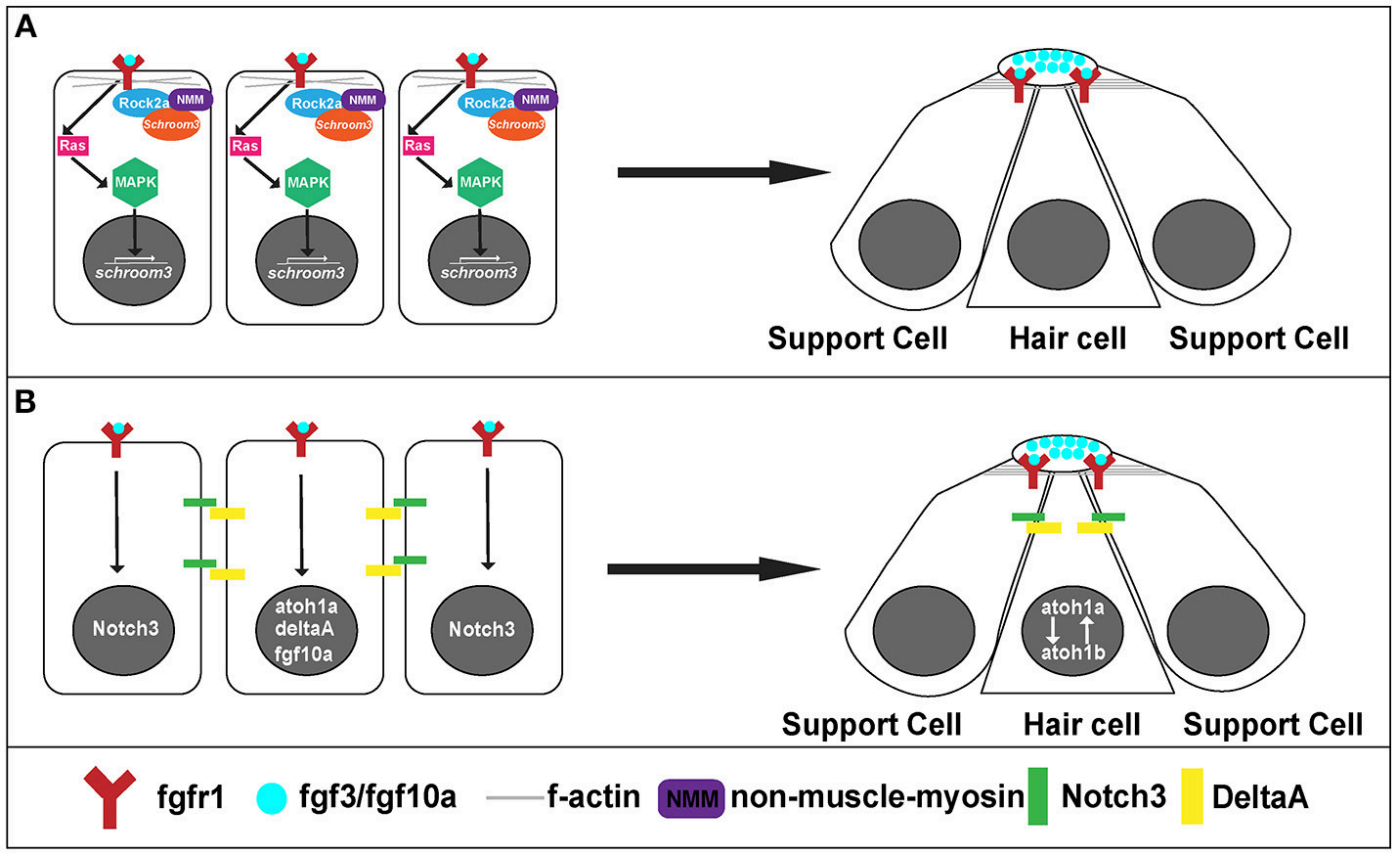
Signaling pathways for rosette formation and proto-NM maturation. **(A)** Hypothesized signaling that initiates apical constriction. Fgf signaling activates Ras and MAPK. This initiates transcription of *schroom3*. Schroom3 interacts with Rock 2a (Rho Kinase) and activates non-muscle myosin at the membrane, which initiates apical constriction of cells through reorganization of the actin cytoskeleton. **(B)** proto-NM maturation signaling. Fgf luminal signaling initiates *atoh1a* and *notch3* expression. Atoh1a induces expression of *deltaA* and *fgf10a*. DeltaA interaction with Notch3 initiates lateral inhibition allowing for *atoh1a* expression to be localized to the central cell (hair cell precursor) and surrounding cells to remain as supporting cells.

Within the pLLP there are a number of signaling pathways that regulate patterning, maintain migratory behavior, and initiate proto-NM differentiation. Among the main pathways are the canonical Wnt signaling pathway (Figure 4; Aman and Piotrowski, 2008), active in the leading region of the pLLP, the Fgf pathway (Figure 4; Lecaudey et al., 2008; Nechiporuk and Raible, 2008), active in the trailing region of the pLLP, and the Notch-Delta pathway active in the forming proto-NM in the trailing region (Figure 5; Matsuda and Chitnis, 2010). Canonical Wnt signaling maintains proliferation, patterning, and migration of the pLLP. Additionally, canonical Wnt signaling is necessary for initiating expression of *fgf3* and *10a* ligands in the leading cells (Aman and Piotrowski, 2008; Matsuda et al., 2013). These ligands activate Fgf signaling in the trailing region and initiate proto-NM differentiation. Wnt signaling also initiates the expression of *dusp6* and *sef* , which are inhibitors of Fgf signaling, allowing for restriction of the Wnt signaling domain to the leading region (Aman and Piotrowski, 2008). Furthermore, Fgf signaling also induces expression of *dkk1* and *dkk2* (Figure 3; Aman and Piotrowski, 2008; McGraw et al., 2014), both of which are inhibitors of Wnt signaling to restrict Fgf signaling to the trailing region. Thus, both Wnt and Fgf mutually inhibit each other to generate region specific signaling domains (Figure 4A).

### Canonical Wnt signaling is necessary for posterior lateral line primordium migration and proto-NM formation

As mentioned above, canonical Wnt signaling is active in the leading third of the pLLP and is necessary for pLLP migration and patterning. Global inactivation of Wnt signaling during pLLP migration causes a loss of proliferation, cell death, and a loss of patterning within the pLLP (Aman and Piotrowski, 2008; McGraw et al., 2011). Additionally, overexpression of Wnt signaling leads to overproliferation and premature termination of the pLLP (Aman and Piotrowski, 2008). Interestingly, exclusive loss of Lef1, a downstream effector of canonical Wnt signaling, causes defects in migration and cellular behavior within the leading region with no effects on cell death or proliferation (McGraw et al., 2011). Specifically, cells in the leading region of the pLLP are prematurely incorporated into NMs and deposited early. This gradual depletion of cells in the leading region ultimately results in a dispersal of the pLLP and an absence of the terminal cluster of NMs (McGraw et al., 2011; Valdivia et al., 2011). In summary, results from many studies highlight the significance of canonical Wnt signaling in regulating multiple cellular behaviors within the pLLP; however, how these behaviors are executed downstream of Wnt is not well understood.

### Fgf signaling is necessary for neuromast formation and differentiation

The Fgf signaling pathway functions downstream of Wnt and is necessary for proto-NM formation (Figure 4A; Lecaudey et al., 2008; Nechiporuk and Raible, 2008; Chitnis et al., 2012). Blocking or reducing Fgf activity inhibits the formation of rosettes and ultimately halts pLLP migration (Lecaudey et al., 2008; Nechiporuk and Raible, 2008; Chitnis et al., 2012;), whereas ectopic expression of Fgf leads to the formation of additional rosettes (Lecaudey et al., 2008).

On a cellular level, Fgf signaling promotes the shape change of epithelial cells from a columnar to an apically constricted morphology during the formation of rosettes or proto-NMs (Figures 3, 4A; Lecaudey et al., 2008; Nechiporuk and Raible, 2008; Chitnis et al., 2012). Two studies published in the same year presented complimentary findings related to the intracellular pathway that drives apical constriction of pLLP cells. In the first study, researchers found that Fgf signaling activates Ras-MAPK signaling which induces Rock2a localization to the apical portion of cells where it activates myosin regulatory light chain and induces apical constriction (Figure 5A; Harding and Nechiporuk, 2012). The second study demonstrated that Fgf signaling transcriptionally regulates Schroom3, a scaffold protein that binds to Rock and has been shown to activate apical constriction in other contexts (Figure 5A; Ernst et al., 2012).

### Neuromast maturation

Neuromasts are the sensory organs that comprise the pLL. These sensory organs are composed of hair cells and supporting cells. Hair cells lie within the middle of the neuromast with support cells surrounding the hair cells. When hair cell bundles are deflected by changes in water current, this information is mechanotransduced through the hair cell and then transmitted back to the brain where it is further processed.

Differentiation into hair and supporting cell precursors occurs during pLLP migration and is driven by Fgf signaling. In addition to its role in apical constriction, Fgf signaling also initiates expression of *atoh1a* (Figure 5B), a transcription factor that is a master regulator of hair cell fate and thus its activation initiates a hair cell program in a small subset (1 to 2 cells) of cells within a forming proto-NM (Sarrazin et al., 2006; Nechiporuk and Raible, 2008). *atoh1a* expression and action is limited to a single focus through Notch-Delta lateral inhibition. *atoh1a* expression induces expression of *deltaA*, whereas expression of its receptor *notch3* is induced by Fgf signaling within the forming proto-NM. Therefore, the DeltaA ligand (driven through the Atoh1a transcriptional program) interacts with the Notch3 receptor (driven by Fgf signaling) on neighboring cells within the proto-NM and inhibits expression of *atoh1a*, in neighboring cells therefore specifying them as supporting cells (Figure 5B; Itoh and Chitnis, 2001; Matsuda and Chitnis, 2010). If Notch3 is blocked, proto-NMs generate more hair cells at the expense of supporting cells (Matsuda and Chitnis, 2010). Through this mechanism, *atoh1a* expression is restricted to the central cell, inducing hair cell progenitor fate in that cell.

Atoh1a also induces expression of both the *fgf10a* ligand and *atoh1b* (Millimaki et al., 2007; Matsuda and Chitnis, 2010) within the same hair cell precursor. Expression of *fgf10a* from the central hair cell progenitor initiates a new Fgf signaling center within the trailing region that promotes maturation of proto-NMs (Figure 5B; Matsuda and Chitnis, 2010). Atoh1b maintains *atoh1a* expression within the central proto-NM and inhibition of Atoh1b results in a reduction in *atoh1a* expression (Millimaki et al., 2007; Matsuda and Chitnis, 2010). Notably, Fgf ligands accumulate in a microlumen at the apical center of the rosette (Durdu et al., 2014). Inhibiting the formation of the microlumen by knockout of Schroom3 results in a reduction of Fgf response in cells that comprise rosettes. This suggests that the microlumen acts to coordinate Fgf signaling among cells of the rosette during migration. In summary, Fgf signaling plays a key role in both the establishment of hair cell precursors as well as maturation of proto-NMs.

### Cell-cell adhesion and cytoskeletal regulation during posterior lateral line primordium migration

During pLLP migration cells remain in a close contact as they cohesively migrate along the trunk of the zebrafish. Cells within the cluster are connected by cadherin mediated adherens junctions. Specifically, E-cadherin and N-cadherin are both expressed in the pLLP but show specific localization within the proto-NM. In the proto-NM, N-cadherin is expressed in both the hair cell progenitor cell (central cell of the proto-NM) and supporting cells, whereas E-cadherin is only expressed in the hair cell progenitor cell (Matsuda and Chitnis, 2010). However, Revenu et al., reported in 2014 that E-cadherin and N-cadherin are expressed in all cells within the pLLP as evidenced by antibody staining against both cadherins (Revenu et al., 2014). Revenu et al. also examined the maturation of adherens junctions using a BAC fluorescent reporter of N-cadherin as N-cadherin shows enhanced localization at apical junctions. Specifically, they reported a role for N-cadherin in initiating the change in morphology from mesenchymal in the leading cells to columnar epithelial in more trailing cells. The authors showed that N-cadherin clusters first and then epithelial columnar reorganization follows. Finally, using tandem fluorescent protein timers, a readout of protein turnover, the authors determined that the N-cadherin localized at apical junctions is more stable than N-cadherin localized at the basolateral membrane. Moreover, the apical junctions become progressively more stable from the leading to the trailing region.

Adherens junctions between cells often trigger activation of intracellular signaling pathways via various binding partners such as catenins. Recently, one of the catenins expressed in the pLLP, Catenin Delta 1, was shown to regulate Rac1 signaling in cultured cells (Mizoguchi et al., 2017). Specifically, mutation of Mib1, an E3 ubiquitin ligase, caused an accumulation of Catenin Delta 1 and hyperactivation of Rac1, which in turn induced ectopic, random non-persistent protrusions and ultimately impaired migration of cultured cells (Mizoguchi et al., 2017). The authors showed that Mib1 is required for pLLP migration and normal protrusive behaviors of pLLP cells. However, it is not clear if Mib1 also regulates Rac1 activity in the pLLP, similar to the *in vitro* model. In fact, not much is known about how Rac1 and other modulators of protrusive activity are regulated during pLLP migration.

Below we will compare mechanisms active during pLLP migration to other examples of collective cell migration and then focus on how collectively invading cancer cells subvert these mechanisms to invade surrounding tissues.

### Distribution of leaders and followers within collectives

Similar to the pLLP, most collectives show division into two different populations of cells, leaders and followers (Figure 1). Leaders are the cells that detect and sense chemotactic cues, exhibit active protrusive behavior, and produce molecular or mechanical cues to guide the trailing population to the proper destination. While leader cells exhibit common behaviors, follower cells have diverse functions and fates depending on the context. For example, follower cells contribute to trachea bronchi or blood vessels during branching morphogenesis whereas during pLLP migration follower cells ultimately contribute the sensory organs of the lateral line system. Leaders and followers show differences in morphology with leaders displaying mesenchymal morphology and in some contexts follower cells displaying polarization. Interestingly, the assignment as leader or follower is not always permanent during this migratory process. In border cell migration for example, the leader cell rotates as the cell with the highest levels of RTK/MAPK signaling acquires the leader cell position (Bianco et al., 2007). Another example in which leader cell identity is not maintained throughout migration is during neural crest cell migration (Kuriyama et al., 2014). Whereas it is not exactly clear what determines neural crest cell leader position, leader cells maintain higher levels of RAC1 activity and exhibit greater protrusive behavior than follower cells (Theveneau et al., 2010). Further, during branching morphogenesis of the trachea in *D. melanogaster*, cells that receive the highest level of the breathless (Fgf) signal take on the role of the leader cell (Caussinus et al., 2008; Lebreton and Casanova, 2014).

Although in many examples of collective cell migration, it is not uncommon for cells to switch positions during migration, this is not observed in wild-type pLLPs. In some instances cells within the pLLP can be forced to move into new positions as a result of experimental manipulation. Haas and Gilmour (2006) found that when wild-type cells were transplanted into *cxcr4b* mutant embryos, they often ended up at the leading edge of the pLLP. Live imaging of chimeric pLLP revealed that this resulted from the tumbling behavior exhibited by wild-type cells in *cxcr4b* mutant pLLPs. They suggested that the tumbling behavior exposes wild-type cells to the Cxcl12a signal and “captures” these cells at the leading edge of the pLLP. Once wild-type cells are captured in the leading region, the pLLP commences normal migration and proper deposition of NMs. A similar tumbling behavior was observed in chimeric primordia that contained *lef1* mutant cells (McGraw et al., 2011). When *lef1* mutant cells were too close to the leading edge (a couple of cell diameters), the chimeric pLLP stopped and tumbled until mutant cells were “pushed back” and excluded from this leading region (McGraw et al., 2011). Once this occurred, the pLLP resumed its migration. The reason for this tumbling behavior is not clear and it is unlikely that this results from *lef1* mutant cells being unable to sense the chemokine, as *lef1* mutants still express normal levels of the *cxcr4b* receptor. Although collectives determine leading and following positions differently, the division of collectives into these two populations allows for cohesive migration of these cohorts of cells.

### Chemotactic cues guide collectives during migration

In order for collectives to migrate in a directional manner they must respond to specific chemotactic cues. Usually, these cues appear as a gradient, with the collective migrating to the highest levels of chemokine or secreted ligand. Leading cells sense these cues and in turn change their behavior, morphology, and protrusive behavior to respond to the cue appropriately (Sutherland et al., 1996; Haas and Gilmour, 2006; Prasad and Montell, 2007). This is often achieved by regulating proteins that remodel the cytoskeleton. For example, RAC1 activation in leader cells in response to guidance cues has been observed in numerous examples of collective cell migration including border cell migration, *D.melanogaster* trachea formation, and neural crest cell migration (Murphy and Montell, 1996; Chihara et al., 2003; Theveneau et al., 2010; Scarpa et al., 2015).

Despite differences in organization of collectives and environmental contexts through which they migrate, some collectives utilize common guidance cues during migration. For example both neural crest cells in *Xenopus* and the cells within pLLP respond to Cxcl12a during migration (Figures 1A, 4B; David et al., 2002; Li et al., 2004; Theveneau et al., 2010). Overexpression of Cxcl12a during neural crest cell migration leads to aberrant migration whereas loss of Cxcl12a during pLLP migration results in inhibition of migration (Valentin et al., 2007; Olesnicky Killian et al., 2009). Additionally, in both of these examples Cxcl12a is uniformly expressed and self-generated gradients are produced by the collectives. During neural crest cell migration, neural crest cells migrate toward the epibranchial placodes, the source of the Cxcl12a (Theveneau and Mayor, 2013). Neural crest cells use contact inhibition of locomotion to facilitate proper migratory behavior. Specifically, neural crest cells extend protrusions that interact with the placodes during the onsent of migration. This induces a repulsive response by placodal cells in which focal adhesions are dissassembled and placodal cells migrate away. Neural crest cells then migrate toward the placodal cells again and migration occurs in a “chase and run” manner (Theveneau et al., 2013).

In the case of pLLP migration, Cxcl12a is expressed uniformly throughout the myoseptum of the zebrafish (Figure 4B; David et al., 2002; Li et al., 2004). In this case, domain specific expression of Cxcl12a receptors, *cxcr4b* and *cxcr7b*, produces a local gradient within the pLLP. *cxcr4b* is expressed in the leading region, whereas *cxcr7b* is expressed in the trailing region. Cxcr4b acts as the chemoreceptor initiating a G-protein signaling cascade within the leading cells whereas expression of *cxcr7b* in the trailing region acts as a ligand sink creating a gradient within the pLLP itself (Haas and Gilmour, 2006; Dambly-Chaudiere et al., 2007; Valentin et al., 2007). These differences in the ligand binding and ligand-receptor turnover lead to a gradient of Cxcl12a response within the pLLP.

Other examples of different guidance cues used during the migration of collectives include EGF and PVF-1, two molecules within the developing *D. melanogaster* oocyte that are necessary for proper border cell migration (Figure 1C). Mutation of either EGFR or PVR results in uncontrolled protrusive behavior and defects in migration (Prasad and Montell, 2007). Finally, Fibroblast Growth Factor (FGF) is used as a chemotactic cue during branching morphogenesis in the trachea, mammary gland, and lung (Figure 1D; Sutherland et al., 1996; Ewald et al., 2008; Metzger et al., 2008) as well as in other examples of collective cell migration such as nephric duct migration, wound healing, and endothelial cell migration (Werner et al., 1992; Vitorino and Meyer, 2008; Attia et al., 2015). In summary, collectives recognize a variety of guidance cues and employ diverse strategies as to interpret these cues and maintain their migratory behaviors.

### Cell-cell junctions and cell-ECM interactions during collective cell migration

In order for cells to migrate cohesively as a group during collective cell migration, cells must communicate often through stable or transient cell-cell junctions. These junctions usually consist of cadherins, desmosomes, and tight junction proteins; loss of these structures often leads to improper or failed migration. Cadherin junctions are the most prevalent junctions observed during collective cell migration. For example, cell-cell junctions between migrating border cells and nurse cells are mediated by the transient presence of E-cadherin and loss of E-cadherin results in decreased protrusion formation at the front of the cluster and ultimately impaired migration (Niewiadomska et al., 1999; Geisbrecht and Montell, 2002; Cai et al., 2014). Additionally, during neural crest cell migration in *Xenopus*, Cadherin-11 is necessary for contact inhibition of locomotion, which allows for processive and directed migration of neural crest cells as described above (Becker et al., 2014). Loss of Cadherin-11 results in non-directional migration and impaired adhesive ability (Becker et al., 2014). In addition to cadherin-based cell-cell junctions, desmosomes and tight junctions are also observed during collective cell migration. In wound healing, both desomosomal-junctions and tight junctions are necessary for proper healing (Danjo and Gipson, 1998; Shaw and Martin, 2009). Knockdown of either desomosomal or tight junction proteins leads to decreases in cell migration velocity and ultimately impairment of migration to close the wound (Bazellières et al., 2015). Desomosomal and tight junctions are also observed during mammary gland morphogenesis. Interestingly, these two junctional complexes show differences in cellular localization within the developing mammary gland (Shamir and Ewald, 2015). Tight junctions are only seen at the apical portion of cells that face lumens, whereas desomosomal junctions connect interior portions of cells in the mammary duct (Shamir and Ewald, 2015).

During migration, cells need to generate force to processively migrate toward their destination. To achieve this, cells adhere to the extracellular matrix as well as use supracellular organization to generate force between the leaders and the followers. To connect with the extracellular matrix cells utilize integrins, which link the intracellular cytoskeleton to the extracellular matrix. This allows transduction of mechanical signals as well as force generation through the recruitment of cytoskeletal adaptor proteins, which couple the integrins and their extracellular binding partners (Nobes and Hall, 1999; Zaidel-Bar et al., 2007). During wound healing, integrin-mediated signaling induces cytoskeletal rearrangements that initiate leader cell properties at the wound edge (Etienne-Manneville and Hall, 2001). These leader cells use the integrins α2β1, α5β1, and αvβ3 to generate force on a collagen substrate and initiate movement to close in the wound (Grose et al., 2002). Similarly, β1 integrins are used during angiogenesis to couple the extracellular matrix to the cytoskeleton within the endothelial collective. β1 integrins activate guanine nucleotide exchange factors (GEFs) for Cdc42 and Rac1 as well as kinases such as Src and FAK (Lamalice et al., 2007; Osmani et al., 2010) to promote protrusive behavior at the leading edge of tip cells, which is necessary for appropriate migratory behavior (Scales and Parsons, 2011; Lawson and Burridge, 2014).

In addition to integrin-mediated force generation, collectives also maintain collective movement and force generation through supracellular cytoskeletal organization. Specifically, focal adhesions at the leading front edge associate with actin-myosin cables, which initiate contraction and force generation. These actin-myosin cables ultimately extend through multiple layers of follower cells allowing for force generation throughout the entire collective instead of the first row of cells (Li et al., 2012; Reffay et al., 2014). However, during collective cell migration the largest forces are generated at the leading edge of collectives with a decrease in force strength in back of the collective (du Roure et al., 2005; Trepat et al., 2009; Tambe et al., 2011; Anon et al., 2012; Cai et al., 2014). For example, during border cell migration, tension decreases at the back of the cluster (Cai et al., 2014). A similar distribution of force is observed in wound healing, where the greatest traction forces are exhibited at the leading edge of the monolayer but forces are maintained among follower cells in the collective (du Roure et al., 2005; Trepat et al., 2009; Tambe et al., 2011; Anon et al., 2012).

In addition to adhering to the extracellular matrix for force generation, cells in collectives also remodel the extracellular matrix while migrating. Migrating and/or surrounding cells deposit new basement membrane to form migrating tracks. The deposition of a new basement membrane forms a smooth surface to promote migration. During neural crest cell migration in both *Xenopus* and chick embryos, neighboring cells deposit fibronectin along neural crest migrating streams to facilitate migration (Alfandari et al., 2003). Similarly, astrocytes underlying the migrating endothelial cells secrete fibronectin during angiogenesis (Stenzel et al., 2011). Fibronectin then induces tip cell filopodia promoting migratory behavior (Stenzel et al., 2011). When fibronectin is specifically deleted from the astrocytes, endothelial cells show defects in migration (Stenzel et al., 2011). In addition, the basement membrane that is produced by endothelial cells and pericytes during angiogenesis helps stabilize the migrating blood vessels (Eming et al., 2007). The deposition of new basement membrane allows for migrating collectives to migrate through the path of least resistance.

### How does collective cell migration influence our understanding of invasive cancer?

**Figure 6:**
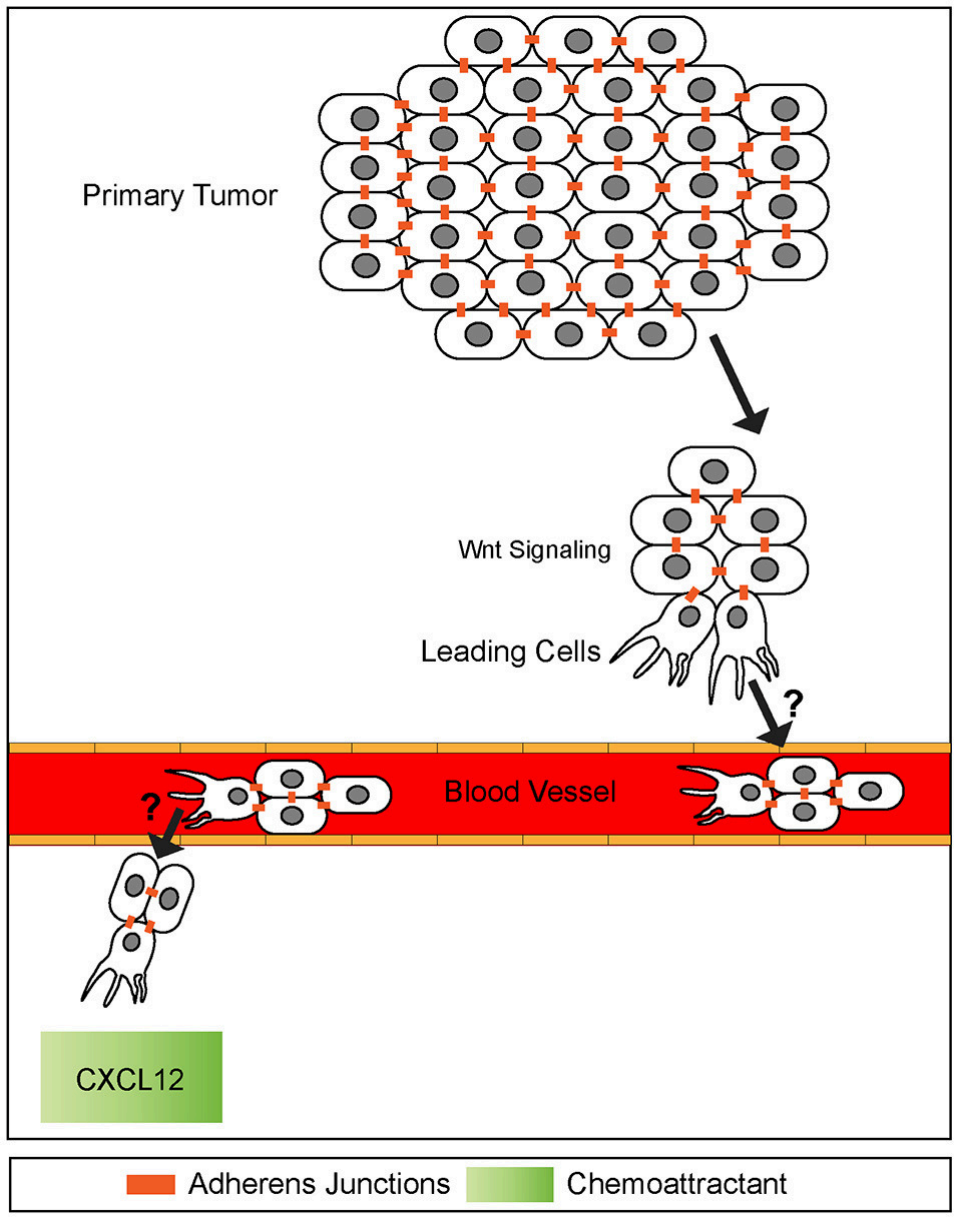
A potential model of collective cell invasion. Small groups of cells high in canonical Wnt signaling break off from the primary tumor, invade surrounding tissue, and enter the blood stream. This cluster then extravisates from the blood stream homing onto to a chemokine source, such as CXCL12. The mechanisms of blood vessel intravisation and extravasation by cell clusters are not known (question marks). It has been speculated that behaviors of such clusters exhibit many similarities to leading front cells present in collective cell migration during embryogenesis. Adherens junctions are maintained throughout invasion process.

The dogma surrounding cancer cell invasion for many years was that single cells would detach from cancerous tumors, enter the blood stream and metastasize in other tissues. However, in the last 50 years that viewpoint has been gradually expanded and it is now widely recognized that in many cases clusters of cells can also detach from the primary tumor to initiate metastasis (Figure 6).

It was first reported more than half a century ago that cancer cell metastasis could be associated with both single and clusters of tumor cells (presumably detached from the primary tumor) found in blood samples of patients (Figure 6) (Zeidman and Buss, 1952). Subsequent research indicated tumor cell clusters were better at initiating metastasis than single cells when intravenously injected into mice (Liotta et al., 1976). Further studies confirmed this observation and revealed that tumor cell clusters are actually 20–30-fold better at initiating metastasis *in vitro* (Cheung et al., 2016) and *in vivo* (Hou et al., 2012; Maddipati and Stanger, 2015; Cheung et al., 2016).

Research over the last two decades strongly suggests that collective cell invasion mediates metastasis in numerous epithelial cancers including prostate, pancreatic, lung, colorectal and breast cancer (Friedl et al., 1995; Nabeshima et al., 2000; Hegerfeldt et al., 2002; Aceto et al., 2014; Gundem et al., 2015; Maddipati and Stanger, 2015; Cheung et al., 2016). However, only recently studies provided most rigorous evidence for collective cell invasion mechanisms in both humans and mouse models. Recent advances in microfluidics and next generation nucleic acid sequencing allowed for isolation and interrogation of a small number of circulating tumor microclusters. In humans, RNA sequencing of circulating cancer cell clusters in comparison to circulating single cancer cells (Aceto et al., 2014) revealed a small subset of differentially expressed genes. One of the transcripts enriched in microclusters was Plakoglobin, a component of desmosomes and adherens junctions. This study revealed that Plakoglobin plays a role in maintaining adherens junctions in cancer cell clusters and thus enhances their metastatic potential. Interestingly, Plakoglobin has been shown to be important in focal adhesions during collective cell migration of mesendoderm in *Xenopus* (Bjerke et al., 2014). Another study investigating breast cancer invasion in a mouse model, found that Keratin14 is upregulated in circulating tumor cell clusters and lung metastasis (Cheung et al., 2016). When Keratin14 was depleted, it disrupted the metastases markers Tenascin C, Jagged 1, and Epiregulin. As Keratin14 is also enriched in desmosomes, this study further emphasizes a critical role for cell-cell adhesions during tumor cluster invasion.

Interestingly, these invasive fronts share molecular similarities to collective cell migration observed during embryonic development. For example, the leading edge of invasive cancer clusters share common molecular properties observed at the leading edges of collectives including cell-cell junctions and cell adhesion receptors (Figure 6) (Freidl et al., 2004; Christiansen and Rajasekaran, 2006; Alexander et al., 2008). In addition, collectively invading cancer cells use self-generated gradients to promote migration. Similar to self-generated gradients by border cells and the pLLP, a study examining invasion of melanoma cells identified a mechanism by which tumor cells generated a gradient of lysophosphatidic acid (LPA) (Muinonen-Martin et al., 2014) and used this gradient to promote cancer cell invasion. In this study, tumor cells acted as a ligand sink by breaking down LPA into byproducts. This created a gradient in which LPA was high in surrounding tissues but low within the tumor itself. Melanoma tumors then used this gradient to migrate to higher sources of LPA in the surrounding tissue, initiating metastasis.

Common signaling pathways used during development as well as collective cell migration are often reactivated during collective invasion (Korc and Friesel, 2009; Katoh, 2017; Bach et al., 2018). For example, many downstream components of the canonical Wnt signaling pathway are misregulated in a variety of cancers (hepatocellular, colorectal, orapharyngeal squamous cell carcinoma) and their associated metastasis including Axins, β-catenin, and TCF/Lef1 transcription factors (Figure 6) (Lammi et al., 2004; Salahshor and Woodgett, 2005; Marvin et al., 2011; Papagerakis et al., 2012). Notably, Lef1 is active at the leading front of invasive lung and colorectal cancers, similar to canonical Wnt signaling being active within the leading region of the pLLP (Nguyen et al., 2009; Wang et al., 2013). In addition to mutations in canonical Wnt signaling, other signaling pathways active during pLLP migration are also implicated in certain types of metastatic cancer. Mutations in the Notch/Delta pathway have been associated with poor prognosis in colorectal and breast cancer as activation of this pathway is associated with metastasis. Although it is not clear whether this pathway is involved in collective cell invasion (Leong et al., 2007; Wang et al., 2013).

Cancer cells also make use of chemotactic signals during metastases. In particular, numerous invasive cancers show abnormal expression of the chemokine receptors CXCR4 and CXCR7 as well as the ligand CXCL12 both within the tumors and at potential sites of metastases. For example in breast cancer, tumor cells express high levels of CXCR4 and metastatic target tissues (lung, liver, bone) express high levels of the ligand CXCL12 (Figure 6) (Wang et al., 2013; Wu et al., 2015). Further, high levels of CXCR4 and CXCR7 are associated with shorter survival times than those with low levels (Wu et al., 2015). Although, this association does not seem to hold true for other types of cancers. In a pancreatic cancer *in vivo* mouse model, cells producing CXCL12 showed deficits in migration and poor metastatic potential in comparison to control cells producing no CXCL12 (Roy et al., 2014). Based on this evidence it is possible that the CXCL12 chemokine may act differently in various cancer contexts. Despite the known prevalence of mutations within these signaling pathways, the mechanisms by which these mutations induce and promote or inhibit collective invasion and metastases remain unknown. Understanding how these signaling pathways regulate collective cell migration of the pLLP may provide clues as to how these pathways are hijacked during cancer invasion.

Based on the similarities between collective invasion and collective cell migration, we can use models of collective cell migration during development to discern mechanisms used by tumor clusters during metastasis. For example, as Lef1 is upregulated at the invasive fronts of both lung and colorectal invasive cancers and canonical Wnt signaling via Lef1 is active in the leading region of the pLLP we can use the leading region of the pLLP as a model for collective cancer invasion. We can study cellular adhesion, protrusive behavior, and cell-ECM interactions using the pLLP model to identify cellular mechanisms that promote cancer front migration and metastasis. Identification of cellular pathways that act downstream of Lef1 in the pLLP may provide clues as to how these factors are misregulated during invasive cancers that show increased Lef1 expression at their leading edge. Thus, further insights gained through studies of pLLP leading edge behavior could provide insight into how these invasive clusters promote metastasis.

## Conclusions

Collective cell migration is a widely used developmental process that initiates and promotes morphogenesis of many different organ systems. While collectives are organized into a variety of different forms, they often share similar cellular strategies. Collectives are guided by leading cells that sense and respond to the extracellular environment, specifically chemotactic cues. These chemotactic cues are then transmitted through specific signaling pathways to initiate molecular changes that guide migration as well as differentiation. Insights gained from studying mechanisms of collective cell migration can be used to identify mechanisms by which invasive cancers hijack developmental machinery to promotemetastasis.

## Author contributions

HO wrote the manuscript. AN edited the manuscript.

### Conflict of interest statement

The authors declare that the research was conducted in the absence of any commercial or financial relationships that could be construed as a potential conflict of interest.

## Abbreviations

pLL: posterior Lateral Line
pLLP: posterior Lateral Line Primordium
NM: Neuromast.
